# Hexosomal Dispersion: A Nano-Based Approach to Boost the Antifungal Potential of Citrus Essential Oils against Plant Fungal Pathogens

**DOI:** 10.3390/molecules26206284

**Published:** 2021-10-17

**Authors:** Mohamed S. Sedeek, Abdulaziz M. Al-Mahallawi, Rania A. A. Hussien, Ahmed M. Abdelhaleem Ali, Ibrahim A. Naguib, Mai K. Mansour

**Affiliations:** 1 Pharmacognosy Department, Faculty of Pharmacy, Cairo University, Kasr-El-Aini Street, Cairo 11562, Egypt; 2 Department of Pharmaceutics and Industrial Pharmacy, Faculty of Pharmacy, Cairo University, Cairo 11562, Egypt; abdulaziz.mohsen@pharma.cu.edu.eg; 3 School of Life and Medical Sciences, University of Hertfordshire Hosted by Global Academic Foundation, New Administrative Capital, Cairo 11562, Egypt; 4 Fungicide, Bactericide and Nematicide Department, Central Agricultural Pesticides Lab (CAPL), Agriculture Research Center (ARC), Giza 11835, Egypt; raniahussien187@gmail.com; 5 Department of Pharmaceutics and Industrial Pharmacy, College of Pharmacy, Taif University, P.O. Box 11099, Taif 21944, Saudi Arabia; a.mali@tu.edu.sa; 6 Department of Pharmaceutical Chemistry, College of Pharmacy, Taif University, P.O. Box 11099, Taif 21944, Saudi Arabia; i.abdelaal@tu.edu.sa; 7 Department of Medicinal Plants and Natural Products, Egyptian Drug Authority, Giza 11553, Egypt; mai.khaled@pharma.cu.edu.eg or

**Keywords:** fungicides, citrus, essential oil, nanotechnology, hexosomes, environmental contamination

## Abstract

The demand for natural fungicides to replace synthetic ones has surged since toxic residues persist in soils, causing environmental contamination and posing a serious threat to worldwide public health. In the context of crop protection and enhancing the efficiency and safety of fungicides, nanotechnology is an eco-friendly strategy in managing fungal pathogens. In the present study, essential oils were isolated from the peels of four citrus fruits (*Citrus lemon*, *Citrus aurantifolia*, *Citrus maxima*, and *Citrus sinensis*) and were investigated using gas chromatography-mass spectrometric analysis. Monoterpene hydrocarbon was the most predominant group and limonene was the most abundant in the four oils. The antifungal potential of the oils was investigated, and the most active oil (*Citrus lemon*) was loaded into hexosomal dispersion, and its antifungal potential was retested against the same fungi. The structurally unique nano-based formulation showed great potency for fungal control. To the best of our knowledge, it is the first time the oil of *Citrus lemon* in nano-hexosomes has been formulated and its fungicidal activity examined. The data collected suggest that citrus essential oils (CEOs), especially when nano-formulated, could be successfully used in integrated fungus management programs.

## 1. Introduction

Fungal plant pathogens play a crucial role in plant production [1]. These pathogens can significantly reduce the productivity and quality of field crops and this is becoming a more pressing concern for human health and the global economy and costs billions of dollars annually [1,2].

Agricultural crops are exposed to more than 10,000 pathogenic fungi that are known to cause important plant diseases [3], resulting in a significant loss in agricultural crops and reduction of world food production. Farmers generally rely on the use of synthetic fungicides to control plant diseases caused by pathogenic fungi; however, misuse of these fungicides can cause serious health and environmental problems [3].

One of the most important fruit crops in the world is citrus [4]. Citrus is grown in over 100 nations throughout the world, primarily in tropical and subtropical regions. As a byproduct of citrus fruit processing, a vast quantity of residual peels is generated, which add no value to the product even though they are discarded or dumped, presenting an environmental problem [5]. Citrus peels have received much attention for their potential use as value-added products because they contain numerous biologically active compounds, including natural antioxidants and essential oils [6,7].

Citrus essential oils (CEOs) have been utilized for therapeutic and health purposes in numerous cultures since ancient times for antibacterial, antiviral, antifungal, anticarcinogenic, antimutagenic, anti-inflammatory, and antioxidant properties [8]. The numerous health benefits linked to the use of CEO have been well-documented. In addition to medicinal and health applications, CEO is increasingly being used in the food sector, food packaging, and agriculture. Synthetic chemical compounds that are more hazardous or to which pests, bacteria, or fungi have proven resistance are being replaced with essential oils [9]. Essential oils have been shown to be more effective than chemical preservatives in reducing pathogen growth and delaying food spoiling in several studies. *Citrus trifoliata* L. essential oil’s insecticidal and fungicidal activities against *Spodoptera littoralis*, *Fusarium oxysporum*, and *Fusarium solani* are examples of citrus essential oils’ potential activity [10,11]. Furthermore, they are free of the negative health hazards connected with synthetic pesticides and fungicides.

Despite their promising features, essential oil-based insecticides have significant limitations linked to their chemical nature (e.g., volatility, poor water solubility, and environmental degradation) [12], which can limit their application. Because of the small size of the particles, encapsulating essential oils inside nanoparticles could alleviate these issues by further enhancing efficacy [11,13]. Essential oils as well as the other botanical products have become a prevalent option for searching new fungicidal agents to be incorporated into the fungus management programs. Nanoencapsulation of fungicidal essential oils would optimize the fungus control system through the effective protection of active ingredients, reducing the need for high doses, the toxicity, and offering protection against environmental degradation and loss. Nanoencapsulation offers significant potential for increasing agricultural productivity while reducing environmental and human health impacts [14,15].

Herein, the fungicidal activity of the essential oil obtained from the peels of *Citrus lemon*, *Citrus aurantifolia*, *Citrus maxima*, and *Citrus sinensis* were assessed against seven pathogenic fungi that have been reported as pathogenic to humans too [16,17,18,19], namely *Rhizoctonia solani*, *Sclerotium rolfsii*, *Fusarium solani*, *Fusarium oxysporum*, *Fusarium semtectium*, *Botrytis cinerea*, and *Alternaria alternata*. In the presented study, a nano-based valorization approach is reported to establish a novel hexosomal system containing the most active essential oil, allowing for an enhancement of the efficiency of natural fungicides, reducing costs and increasing competitiveness.

## 2. Results and Discussion

### 2.1. Chemical Composition of Different Citrus Peels’ Essential Oils

The results obtained by GC-MS analysis of *C. lemon*, *C. aurantifolia*, *C. maxima*, and *C. sinensis* peel essential oils are presented in Table 1. Twenty-four compounds were identified, constituting 97.66%, 94.93%, 99.99%, and 99.93% of the citrus peels essential oils under investigation, respectively. Analysis of C. maxima and *C. sinensis* oil revealed that most of the compounds were monoterpenes, where limonene *represented* the major one, with the percentages (97.51% and 96.71%) in agreement with previously published data [11,20]. *C. lemon* and *C. aurantifolia* essential oils’ composition showed that the percentage of monoterpenes is 64.13% and 80.34%, respectively. The oxygenated hydrocarbon composition is higher in *C. lemon* and *C. aurantifolia*, with percentages of 33.53% and 14.59%, respectively. High *concentrations* of oxygenated hydrocarbons in *C. lemon* and *C. aurantifolia* could be attributed to the strong antifungal activities of these oils [21]. They could synergistically increase the effect of limonene and other monoterpene hydrocarbons [22].

### 2.2. Preparation and Characterization of Nano-Hexosomal Dispersions

**Figure 1:**
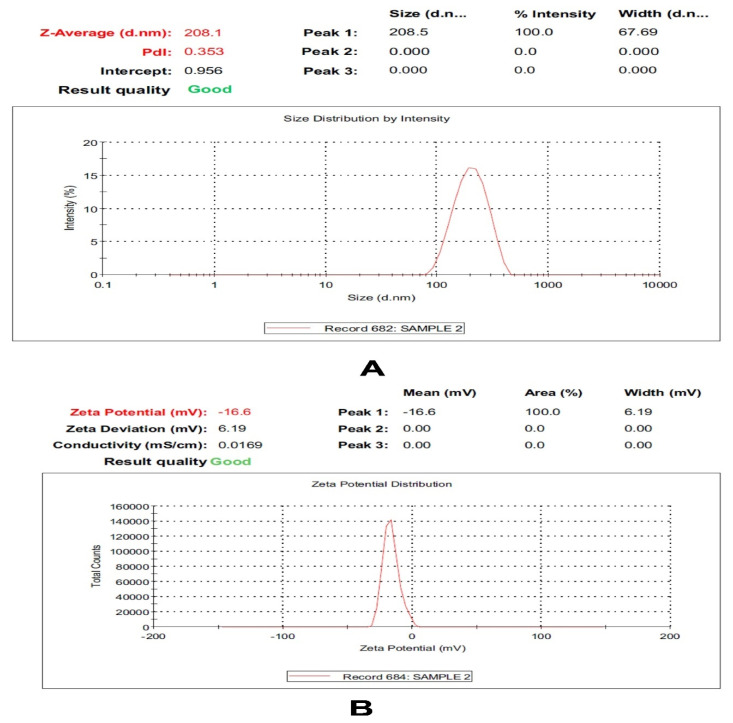
Particle size distribution and zeta potential of *Citrus lemon* essential nanohexosomes.

**Figure 2:**
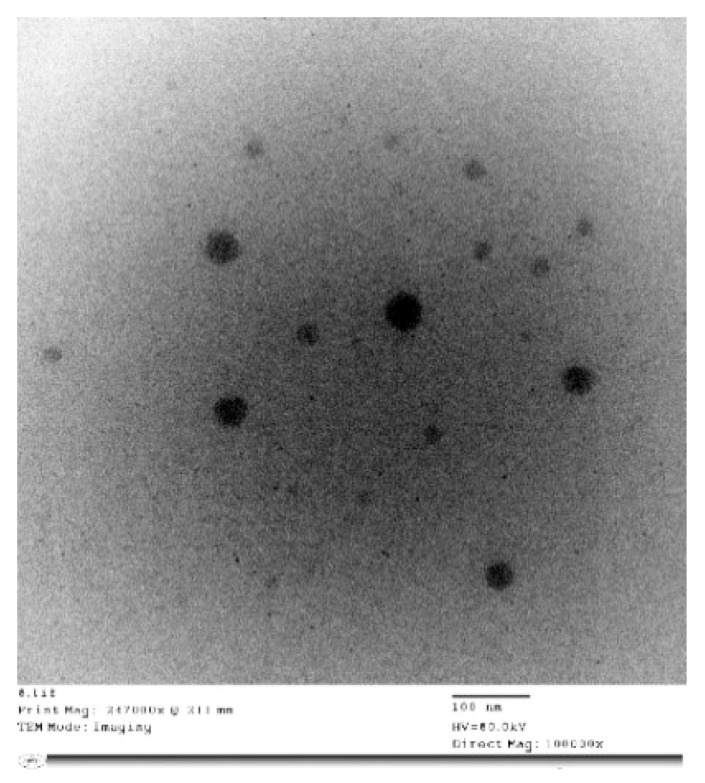
Transmission electron micrographs of different nano-hexosomal dispersions of *C. lemon*.

The hexosomal system with its unique architecture is a promising nanoplatform that is capable of being loaded with hydrophobic and hydrophilic active molecules. In the current study, the hexosomal system was successfully prepared by the hot emulsification method. The melted lipids (glyceryl monooleate (GMO)/oleic acid together with volatile oil) were emulsified into an aqueous phase containing a stabilizer (Pluronic F127) at 70 °C. The presence of oleic acids changes the GMO effective packing parameter that induces the phase transition from the cubic phase (cubosomes) to the hexagonal phase (hexosomes). The prepared volatile oil-loaded hexosomal system showed a particle size (PS) in the nano-range (210.35 ± 3.18 nm) and acceptable polydispersity index (PDI) values (0.31 ± 0.0.6). Moreover, the prepared system demonstrated a negative and zeta potential (ZP) value (−16 ± 0.84 mV), as shown in Figure 1. This negative surface charge is probably attributed to the presence of a carboxylic end group in the fatty acid, oleic acid [23]. A representative transmission electron microscope (TEM) of the volatile oil-loaded hexosomal system is illustrated in Figure 2, where nearly hexagonal nonaggregated particles were observed.

### 2.3. Antifungal Activity of Different Citrus Oil and Nano-Hexosomal Formula of the Most Active One

**Figure 3:**
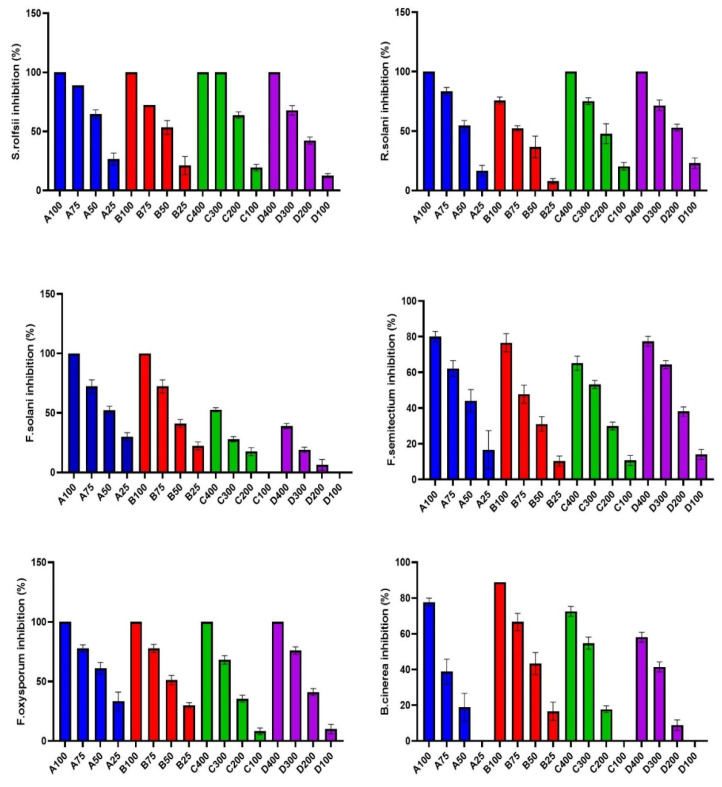
Mycelial growth % inhibition with different concentrations (µL/mL) of citrus essential oils (A: *C. lemon*, B: *C. aurantifolia*, C: *C. maxima*, D: *C. sinensis*) against different phytopathogenic fungi.

Different citrus peel essential oils (*C. lemon*, *C. aurantifolia*, *C. maxima*, and *C. sinensis*) were assessed against different phytopathogenic fungi (*Rhizoctonia solani*, *Sclerotium rolfsii*, *Fusarium solani*, *Fusarium oxysporum*, *Fusarium semtectium*, *Botrytis cinerea*, and *Alternaria alternata*). All tested CEOs exerted strong antifungal activity in a dose-dependent manner where percentage inhibition increased with higher doses, as illustrated in Figure 3. *C. lemon* and *C. aurantifolia* essential oils with a concentration of 100 µL/mL showed complete inhibition of *S. rolfsii*, *F. solani*, and *F. oxysporum* mycelium growth, as shown in Figure 3. The lowest IC_50_ was recorded with *F. oxysporum* (36.92, 41.72), *S. rolfsii* (37.59, 45.60), and *F. solani* (42.17, 50.79 µL/mL) in case of *C. lemon* and *C. aurantifolia*, respectively. On the other hand, *B. cinerea* was more tolerant to *C. lemon* with a high IC_50_ (78.60 µL/mL) compared to the other fungi. From the above results, it is evident that *C. lemon* and *C. aurantifolia* exert potent fungi toxic effects. 

The results in Table 2 and Figure 3 indicate that the treatment with *C. maxima* and *C. sinensis* reduced the linear growth of all tested phytopathogenic fungi at concentrations of 100, 200, 300, and 400 µL/mL. They exerted complete inhibition of mycelium growth for *S. rolfsii, R. solani,* and *F. oxysporum* with a concentration of 400 µL/mL, as represented in Figure 3. *R. solani* was more resistant to *C. maxima* and *C. sinensis*, the IC_50_ of which was the lowest concentrations (191.1 and 180 µL/mL), respectively, compared to the other fungi. On the other hand, *F. solani* was more tolerant to *C. maxima* and *C. sinensis*, the IC_50_ of which was highest concentrations (395.9 and 457.9 µL/mL), respectively, compared to the other fungi. Their potent antifungal activity could be related to the high concentration of monoterpenes, especially limonene [20,24]. CEOs showed a strong fungi toxic effect through damage and loss of integrity, the rigidity of the cell wall and retraction of cytoplasm in the hyphae, and finally, death of the mycelium as reported in previous studies [25,26]. *C. lemon* essential oil is the only one that inhibited the mycelial growth of Alternaria alternata, with IC_50_ of 229.1 µL/mL. *C. lemon* essential oil is the most active oil with a high concentration of oxygenated compounds that could synergize the antifungal effect of monoterpene hydrocarbons [27]. The nano-hexosomal formula was prepared from the most active one (*C. lemon* essential oil), to boost its antifungal activity and allow the production of a natural potent pesticide drug against dangerous phytopathogenic fungi that affect many commercial crops and plants. 

**Figure 4:**
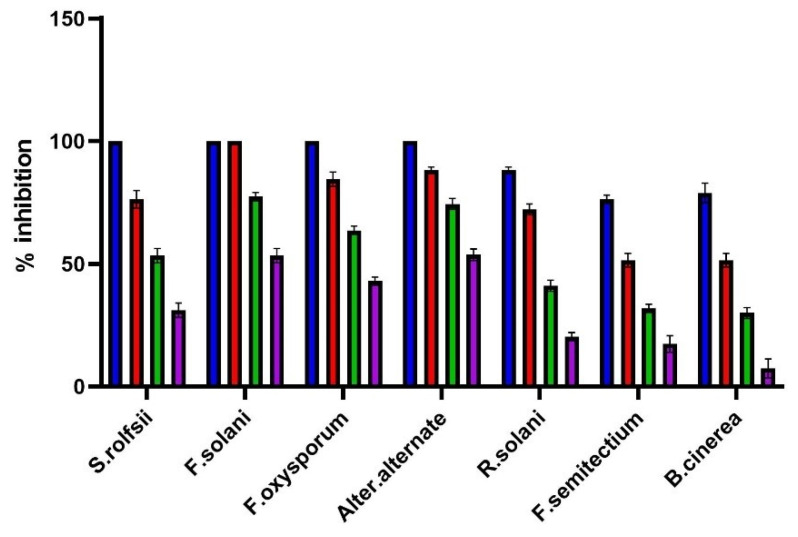
Mycelial growth % inhibition of *Citrus lemon* nanohexosomal formula.

**Figure 5:**
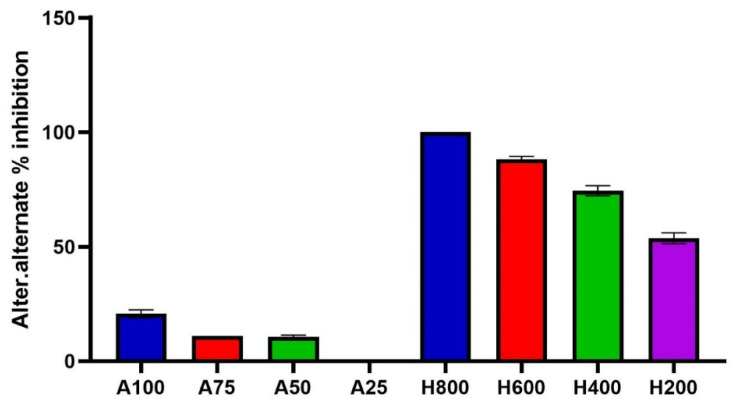
Mycelial growth % inhibition of *Citrus lemon* essential oil and its nano-hemosome formula.

The treatment *potato dextrose agar* (PDA) medium with nano-hexosomes of *C. lemon* essential oil at different concentrations showed a potent antifungal effect against different phytopathogenic fungi under investigation, as illustrated in Figure 4. It was the most effective in inhibiting the mycelial growth of F. solani, which reached 100% inhibition at 600 µL/mL, while at 800 µL/mL, it completely inhibited the mycelial growth in the case of *S. rolfsii*, *F. oxysporum*, and A. alternata. Nano-hexosomes have a moderate effect on *R. solani, B. cinerea*, and *F. semitectium*, with IC_50_ of 416, 549.4, and 534 µL/mL, respectively. Nano-hexosomes of *C. lemon* essential oil showed potent activity against A. alternata, with IC_50_ of 95.54 µL/mL. Although CEOs did not inhibit A. alternata growth, only *C. lemon* weakly inhibited it, as shown in Figure 5. The current study proved that nano-hexosomes are among the most cost-effective drug-loaded lipid-based system that delivers essential oils in their bioactive form. CEO represent only 10% of the nano-hexosomes formula, so a low amount of oil is needed; thus, it is commercially useful to use this formula instead of chemical pesticides that show toxicity and harm to humans. Essential oils are composed of volatile constituents and undergo enzymatic reactions that decrease their activity and limit essential oil use [28]. Encapsulation of essential oil in a drug delivery system via nanotechnology overcomes the previously mentioned problems and improve its stability, bioavailability, and biological activities [11,28]. So, the CEOs nanohexosomes in our study potentiate the antifungal activity of the oil. 

## 3. Materials and Methods

### 3.1. Materials

Fruits of *Citrus lemon*, *Citrus aurantifolia*, *Citrus maxima*, and *Citrus sinensis* were collected from Horticulture Research Institute, Giza, Egypt. The plant’s authenticity was generously validated by Mrs. Therese Labib, Botanical Specialist and consultant at Orman and Qubba Botanical Gardens, Giza, Egypt. Glyceryl monooleate (GMO), oleic acid, and Pluronic F127 (MW: 12,600 Da) were purchased from Sigma- Aldrich.

### 3.2. Methods

Preparation of the essential oil from fruits.

Peels of the fruits were washed, dried, and powdered mechanically. Their essential oils were extracted by hydro-distillation in a Clevenger’s apparatus for 5 h according to the procedure described in the Egyptian Pharmacopeia [29]. The essential oils were dried with anhydrous sodium sulphate and stored in amber glass vials at 4 °C for use in further chemical and biological studies.

#### 3.2.1. GC-MS Analysis and Quantification

Mass spectra were recorded using Shimadzu GCMS-QP2010 (Tokyo, Japan) equipped with a Rtx-5MS fused bonded column (30 m × 0.25 mm i.d. × 0.25 μm film thickness) (Restek, Bellefonte, PA, USA) equipped with a split–splitless injector. The capillary column was coupled to a quadrupole mass spectrometer (SSQ 7000; Thermo-Finnigan, Bremen, Germany). The initial column temperature was held at 45 °C for 2 min (isothermal) and programmed to 300 °C at a rate of 5 °C/min and kept constant at 300 °C for 5 min (isothermal). The injector temperature was 250 °C. The helium carrier gas flow rate was 1.41 mL/min. All the mass spectra were recorded applying the following conditions: (equipment current) filament emission current, 60 mA; ionization voltage, 70 eV; ion source, 200 °C. Diluted samples (1% *v*/*v*) were injected with split mode (split ratio 1: 15). The sample (1 μL) was injected automatically into the chromatograph using an AOC-20i auto-sampler. Volatile components were deconvoluted using AMDIS software (www.amdis.net) and identified by its mass spectrum matching to the database and with authentic standards (when available) [30].

#### 3.2.2. Preparation of Hexosomal Dispersion

Hexosomal dispersions loaded with volatile oils were prepared using a hot emulsification method as described by Abdel-Bar et al. [31], with slight modifications. In brief, GMO (1 g), oleic acid (0.5 g), and volatile oil (1 g) were weighed accurately in a glass vial and allowed to melt at 70 °C. Pluronic F127 (0.5 g) was dissolved in deionized water (7 g) at the same temperature. The molten lipid phase was slowly added into the aqueous phase and homogenized for 5 min at 70 °C. The final milky dispersion was allowed to cool gradually to room temperature. It was kept in glass vials at 2–8 °C for further studies. The final concentrations of the hexosomes’ components (GMO, oleic acid, volatile oil and Pluronic F127, and deionized water) were 10, 5, 10, 5, and 70% *w*/*w*, respectively. 

#### 3.2.3. Measurement of Particle Size, Polydispersity Index, and Zeta Potential

The average particle size (PS) and size distribution expressed as the polydispersity index (PDI) were estimated by dynamic light scattering (DLS) at 25 °C using a Zetasizer Nano ZS (Malvern Instruments, Malvern, UK) [32,33]. The zeta potential (ZP) was determined by the same instrument [34,35]. Prior to performing the determinations, all dispersions were appropriately diluted (100 times) using distilled water. Triplicate measurements were always obtained for each determined response [36,37].

#### 3.2.4. Transmission Electron Microscopy (TEM)

The morphology of the volatile oil-loaded hexosomal system was envisioned via TEM (Joel JEM 1230, Tokyo, Japan). A copper grid was loaded with the diluted dispersion, which was subjected to negative staining with aqueous solution of phosphotungstic acid (2% *w*/*v*) for a duration of 5 min. Drying of the grid at ambient temperature for 10 min was then followed prior to visualization under a transmission electron microscope [35].

#### 3.2.5. Fungal Strains

Cultures of plant pathogenic fungi (*Rhizoctonia solani*, *Sclerotium rolfsii*, *Fusarium solani*, *Fusarium oxysporum*, *Fusarium semtectium*, *Botrytis cinerea*, and *Alternaria alternata*) were provided by fungicide, Bactericide and Nematicide Department, Central Agricultural Pesticide Laboratory (CAPL). Each fungus was maintained on potato dextrose agar (PDA) and stored at 5 °C for further studies.

#### 3.2.6. Antifungal Assay

The antifungal activity of CEOs and hexosomal dispersion of *C. lemon* was determined by the food poisoned technique [38,39]. Different concentrations of CEOs (µL/mL) were mixed with 50 mL of sterilized PDA medium and transferred equally into three petri dishes. The media was allowed to solidify. Then, a seven-day-old fungal culture disk with a 6 mm diameter was taken and inoculated to the center of the petri dishes containing plant extracts. PDA medium without plant extract served as a control. All dishes were incubated at 27 ± 2 °C and the radial growth of colonies was measured when the mycelia of the control had almost filled the petri dishes. Each test was performed in triplicate. 

The fungal growth inhibition was calculated according to the treatment against the control using the following formula [40]:

% inhibition = C − T/C × 100(1)

where C is the average of three replicates of hyphal extension (mm) of the control and T is the average of three replicates of hyphal extension (mm) of plates treated with the tested material.

IC_50_ was calculated as the concentration of the tested compound that decreases the mycelial growth by half between the base and the maximum.

#### 3.2.7. Statistical Data Interpretation

Data analysis and graphs were made using the GraphPad Prism Version 9 program. The data are expressed as an arithmetic mean, standard deviation, and 95% confidence interval for the IC_50_ parameter. P values less than or equal to 0.05 were considered statistically significant.

## 4. Conclusions

Results of the antifungal screening in this study support the recommendation of authors to use CEOs obtained from the peels of *C. lemon*, *C. aurantifolia*, *C. maxima*, and *C. sinensis* as a first defense line against a wide spectrum of phytopathogenic fungi. The antifungal potential of *C. lemon* and *C. aurantifolia* could be attributed to the synergetic effect of their oxygenated hydrocarbons together with the limonene content. CEOs as natural fungicides are considered valuable sources for fungal control without the toxicity and the health hazards connected with synthetic fungicides. Besides, their use in crop management protocols aids in managing the residual peels generated during citrus fruit processing, which solves an environmental problem and compensates for economic losses. The introduction of the most active essential oil (*C. lemon*) into a hexosomal dispersion was performed to create a potential nano-fungicide against plant fungal pathogens. The nano-hexosomes of *C. lemon* essential oil exhibited powerful fungicidal activity that exceeded the oil itself specially against *A. alternata*. The presented results indicate that the nano-hexosomal dispersion helped to boost the antifungal properties of the oil and can be used as a natural nano-fungicide in plant pathogen control. Concisely, it is considered a potential carrier for enhancing the fungicidal activity of CEOs and can be used in integrated fungus management programs.

## Figures and Tables

**Table 1:** Essential oil composition of *C. lemon*, *C. aurantifolia*, *C. maxima*, and *C. sinensis*.

No.	RT	RI	Identified Compounds	Area Percentage			
				*C. lemon*	*C. aurantifolia*	*C. maxima*	*C. sinensis*
1	7.344	948	α-Pinene	0.83	0.72	0.43	0.29
2	7.772	955	Camphene	0.23	–	–	–
3	8.643	982	β-Pinene	11.15	7.63	0.49	–
4	9.086	991	β-Myrcene	0.87	0.79	1.24	1.05
5	10.126	998	p-Cymene	1.14	0.88	–	–
6	10.360	1018	D-Limonene	44.36	61.89	97.51	96.71
7	11.189	1063	γ-Terpinene	2.91	7.50	–	–
8	12.098	1079	α-terpinolene	1.53	0.93	–	–
9	12.471	1082	Linalool	1.69	2.14	0.09	1.53
10	12.615	1097	Nonanal	0.17	–	–	–
11	12.905	1098	Fenchol	0.39	0.14	–	–
12	13.856	1121	Camphor	–	–	–	0.24
13	14.579	1125	p-Mentha-1,5-dien-8-ol	1.02	0.33	–	–
14	14.878	1137	Terpinen-4-ol	2.79	2.03	–	–
15	15.307	1143	α-Terpineol	9.95	6.43	0.23	0.11
16	16.793	1174	β-Citral	13.51	2.52	–	–
17	16.904	1190	Carvone	3.53	0.68	–	–
18	17.198	1228	cis-Geraniol	0.48	–	–	–
19	17.815	1268	Perillaldehyde	–	0.32	–	–
20	20.895	1339	β-Bourbonene	0.13	–	–	–
21	21.845	1410	Caryophyllene	0.13	–	–	–
22	23.475	1515	Germacrene D	0.1	–	–	–
23	24.115	1518	cis-α-Bisabolene	0.64	–	–	–
24	25.439	1603	Germacrene B	0.11	–	–	–
Percentage of identified constituents				97.66	94.93	99.99	99.93
Percentage of identified hydrocarbons				64.13	80.34	99.67	98.05
Percentage of oxygenated hydrocarbons				33.53	14.59	0.324	1.88

**Table 2:** IC_50_ of Citrus essential oils and hexosomal dispersion of *C. lemon* against different phytopathogenic fungi.

Plant Oil	IC_50_ (µL/mL)						
	*Rhizoctonia solani*	*Sclerotium rolfsii*	*Fusarium solani*	*Fusarium oxysporum*	*Fusarium semtectium*	*Botrytis cinerea*	*Alternarai alternata*
***Citrus lemon***	45.29	37.59	42.17	36.92	55.92	78.60	229.10
***Citrus aurantifolia***	66.52	45.60	50.79	41.72	70.03	53.56	0
***Citrus maxima***	191.10	159.00	395.90	232.70	290.70	294.80	0
***Citrus sinensis***	180.50	217.40	457.90	216.20	236.70	351.40	0
***Citrus lemon* nano-hexoosome**	416.00	324.90	193.70	124.30	534.00	549.40	95.54

## Data Availability

Data by authors are available upon request.

## Sample Availability

No new compounds emerged in this study.
